# Use of intravoxel incoherent motion imaging to monitor a rat kidney chronic allograft damage model

**DOI:** 10.1186/s12882-019-1545-1

**Published:** 2019-10-10

**Authors:** Qiang Zhang, Zexing Yu, Song Zeng, Lu Liang, Yue Xu, Zijian Zhang, Hao Tang, Wenjiao Jiao, Wenrui Xue, Wei Wang, Xiaodong Zhang, Tao Jiang, Xiaopeng Hu

**Affiliations:** 1 Department of Urology, Beijing Chao-Yang Hospital, Capital Medical University, NO.8 GongTi South Road, Beijing, 100020 China; 2 Department of Ultrasonography, Beijing Chao-Yang Hospital, Capital Medical University, NO.8 GongTi South Road, Beijing, 100020 China; 3 Department of Radiology, Beijing Chao-Yang Hospital, Capital Medical University, NO.8 GongTi South Road, Beijing, 100020 China; 4 Department of Urology, Beijing YouAn Hospital, Capital Medical University, NO.8 Youanmenwai Xitoutiao, Beijing, 100069 China

**Keywords:** Chronic allograft damage, Graft fibrosis, Intravoxel incoherent motion imaging, Diffusion-weighted imaging

## Abstract

**Background:**

Chronic allograft damage (CAD) is the leading cause of long-term graft dysfunction. A noninvasive method that can diagnose CAD early and monitor its development is needed.

**Methods:**

Kidneys from Fisher rats were transplanted into Lewis rats to establish a CAD model (*n* = 20). The control group underwent syngeneic kidney transplantation (*n* = 20). The serum creatinine of the rats was monitored. At 4, 12, and 20 weeks after modeling, a magnetic resonance imaging (MRI) examination was performed. The apparent diffusion coefficient (ADC), pseudo diffusion coefficient (D*), true diffusion coefficient (D) and perfusion fraction (f) of the two groups were analyzed. Chronic allograft damage index (CADI) scoring was used to evaluate the transplanted kidney specimens. Immunohistochemistry was used to detect the expression of fibrosis markers in the transplanted kidney tissues and to analyze their correlations with all MRI parameters.

**Results:**

The transplanted kidneys in the experimental group developed CAD changes before the appearance of elevated creatinine. The MRI parameters in the experimental group [ADC (1.460 ± 0.109 VS 2.095 ± 0.319, *P* < 0.001), D (1.435 ± 0.102 VS 1.969 ± 0.305, *P* < 0.001), and f (26.532 ± 2.136 VS 32.255 ± 4.013, *P* < 0.001)] decreased, and D* (20.950 ± 2.273 VS 21.415 ± 1.598, *P* = 0.131) was not significantly different from those in the control group. ADC, D and f were negatively correlated with the CADI and the α-SMA and vimentin expression levels.

**Conclusion:**

Intravoxel incoherent motion **(**IVIM) imaging could detect CAD earlier than creatinine and reflect the degree of fibrosis in grafts quantitatively.

## Background

Kidney transplantation is the best alternative treatment regimen for end-stage renal disease (ESRD). Although the short-term efficacy of kidney transplantation has significantly increased, long-term graft survival has not significantly improved. The main factor affecting the long-term survival of transplanted kidneys is chronic allograft damage (CAD) [1]. When the transplanted kidney develops rejection, the graft is injured, and collagen fibers continue to accumulate, resulting in interstitial fibrosis of the graft. In addition, the continuous progression of fibrosis will eventually cause transplanted kidney failure [2]. Currently, no effective treatment is available for CAD after its occurrence. However, early discovery and timely intervention can block further damage to the graft [3]. At present, the gold standard for the diagnosis of CAD is a graft biopsy. However, usually patients are reluctant to accept this procedure because of its invasiveness and implementing this approach in clinical practice is difficult [4]. Thus, early noninvasive diagnosis of CAD has become an important issue to be resolved in the transplantation field.

Magnetic resonance imaging (MRI) has the advantages of noninvasiveness, ease of operation and excellent repeatability; therefore, its value in clinical practice has been recognized by the medical field. Functional magnetic resonance imaging (fMRI) not only can display changes in the anatomical morphology of the kidneys but also can be used to noninvasively and quantitatively monitor their function and pathophysiological changes [5, 6]. Intravoxel incoherent motion (IVIM) imaging can obtain parameters such as the pseudo diffusion coefficient (D*), true diffusion coefficient (D) and perfusion fraction (f) to quantitatively evaluate blood flow changes in parenchymatous organs [7, 8].

This study aimed to explore the value of IVIM imaging for the early diagnosis of CAD and to evaluate the degree of fibrosis in rat model.

## Methods

### Animals and reagents

This study was approved by the Ethics Committee of Capital Medical University (approval: AEEI-2017-013). The experimental animals were 8–12 weeks old, specific pathogen-free (SPF) grade, male, inbred Fisher and Lewis rats with body weights of 200–250 g. The rats were provided by Beijing Vital River Laboratory Animal Technology Co., Ltd. (permit number: SCKX Jing 2012–0001). The animals were housed at 22 ± 1 °C at a relative humidity of 55 ± 5% under 12 h light-dark cycles and were fed a standard rat diet. The animals were under the protection of the U.K. Animals (Scientific Procedures) Act 1986 and associated guidelines (EU Directive 2010/63/EU) for animal experiments. The Guide for the Care and Use of Laboratory Animals (NRC 2011) was also obeyed. The hematoxylin and eosin (HE) staining reagent kit, periodic Schiff-methenamine silver (periodic acid-silver methenamine, PASM) staining solution, and Masson’s trichrome staining solution were purchased from Techlab Biotechnology Co., Ltd. (Beijing, China). The anti-alpha smooth muscle actin (α-SMA) and anti-vimentin antibodies were purchased from Abcam Limited Corporation (Cambridge, UK).

### Establishment of the animal model [9]

A total of 20 Fisher and 20 Lewis rats were used to establish the CAD model in the experimental group. The following animal experiments were carried out in animal laboratory at night. The rats were anesthetized with isoflurane in oxygen. General anesthesia was induced with 3% and maintained with 1.5% isoflurane. The left kidney of healthy Fisher rats was used as the donor kidney. The left kidney of Lewis rats was resected, and orthotopic transplantation of the donor kidney was performed on the left side. The rats received subcutaneous injections of low-dose cyclosporine at a dose of 1.5 mg/kg/day on the day of surgery and days 1–10 after surgery. The right original kidney was resected 7 days after surgery. In the control group, 40 Lewis rats were used to establish the orthotopic syngeneic kidney transplantation model. The same anesthesia procedure was adopted, and male Lewis rats in the same litter were used as the donors and recipients. After surgery, the same treatment was performed as described for the experimental group. The donor rats were euthanized by adjusting the CO_2_ flow rate in the cage to 5 L/min until one minute after breathing stopped.

### Measurement of transplanted kidney functions

Tail vein blood samples were extracted from all rats for assessment of transplanted kidney function. The measurement was performed once every 2 weeks. The blood creatinine concentration (mmol/L) was measured using the picric acid method with the Dimension Rxl Max automatic biochemical analyzer (Siemens, Newark, DE, USA).

### MRI examination of the transplanted kidneys

#### Prescanning preparation and equipment

All rats underwent fasting for 12 h and water deprivation for 4 h before scanning. After anesthetized with 3% isoflurane and maintained with 1.5% isoflurane, the rats were placed in the supine position with all 4 limbs immobilized. A Siemens Prisma 3.0 T superconducting scanner with a 64-channel head-coil system (MAGNETOM Prisma System, Siemens Medical Solutions, Erlangen, Germany) was used.

#### Scanning sequences and parameters

The following images were acquired: coronal and transverse T2-weighted fast spin-echo sequence (TSE), coronal T1-weighted TSE and transverse echo-planar IVIM and diffusion weighted images (DWIs). The parameters for coronal position TSE were as follows: slice thickness, 2.0 mm; slice interval, 0.2 mm; ① T1 turbo spin-echo (TSE): repetition time (TR), 650 ms; echo time (TE), 10 ms; field of view (FOV), 120 mm × 120 mm; band width, 250 Hz/Px; matrix, 256 × 256; and number of excitations (NEX), 4.0; and ② T2 TSE: TR, 3460 ms; TE, 35 ms; FOV, 120 mm × 120 mm; band width, 250 Hz/Px; matrix, 256 × 256; and NEX, 4.0. The parameters for the axial position T2 TSE were as follows: slice thickness, 2.0 mm; slice interval, 0.2 mm; TR, 650 ms; TE, 10 ms; FOV, 120 mm × 120 mm; band width, 250 Hz/Px; matrix, 256 × 256; and NEX, 4.0. The IVIM was performed using the following parameters: TR, 2100 ms; TE, 44 ms; FOV, 120 mm × 100 mm; band width, 1500 Hz/Px; matrix, 120 × 84; and NEX, 1.0. The b values were 0, 50, 100, 150, 200, 400, 800 and 1000 s/mm^2^, which were added to the three orthogonal directions to reduce the influence of diffusion anisotropy. The total acquisition time of each IVIM was 4 min and 40 s. DWI was performed using the following parameters: TR, 2100 ms; TE, 44 ms; FOV, 120 mm × 100 mm; band width, 1500 Hz/Px; matrix, 120 × 84; NEX, 1.0; and b = 0, 800.

#### Image postprocessing and analysis

T2 images were used for analysis of location, morphology and signal changes of the transplanted kidneys. The Siemens Syngovia postprocessing workstation was used to transfer DWI raw data and measure the values of the apparent diffusion coefficient (ADC). The Medical Imaging Interaction Toolkit (MITK, www.mitk.org) was used to obtain all IVIM parameters (D, D*, and f). The kidney upper, mid and lower poles were randomly selected and the region of interest (ROI) was chosen in the renal cortex on the axial position. Blood vessels and the renal pelvis and calyx were avoided. The size of each ROI was completely consistent at approximately 1~2 mm^2^, and at least 5 pixels were included (Fig. 2). Finally, the mean value of 2 observers was used as the value of the parameter for the data analysis. Data were independently collected by researchers blinded to the group identity.

### Pathological analysis of the transplanted kidney specimens

After the MRI examinations, 5 rats from each group were randomly selected using the random number table method at postoperative weeks 4 and 12. All rats were euthanized using the same method described for the donor rats. After the rats were sacrificed, the kidneys were removed immediately, the kidney capsule was removed, and the kidneys were thoroughly washed with ice-cold normal saline. The transplanted kidneys were longitudinally dissected from the coronal plane of the renal hilum and placed in a 10% neutral-buffered formalin solution to fix for 24 h. All acquired kidneys were embedded in paraffin, sectioned (3-μm thickness) and stained with HE, Masson’s stain and PASM. Immunohistochemistry-stained sections were also fixed, embedded and sectioned using the same methods. The sections were placed in 3% H_2_O_2_ to block endogenous peroxidase, followed by pH 6 citric acid buffer, heated at 98 °C in a microwave oven for 15 min, and then cooled for antigen retrieval. The primary antibody was dropped onto the sections (1100 antibody dilution), incubated at 4 °C overnight and washed with phosphate-buffered saline (PBS) for 5 min × 3 times. Each section was incubated with 50 μL of horseradish peroxidase (HRP)-labeled avidin delivered in a dropwise manner, incubated at 37 °C for 30 min and washed 3 times with PBS. Each section received freshly prepared diaminobenzidine (DAB) solution in a dropwise manner, and the reaction was observed and controlled under a microscope. After the nuclei were stained, the sections were mounted using neutral balsam.

Routine pathological sections and immunohistochemistry-stained sections were independently observed under a microscope by 2 senior pathologists. Routine pathological sections of kidney specimens were scored using the chronic allograft damage index (CADI). The CADI scoring system includes 6 sections of CAD pathology (renal interstitial inflammation, renal interstitial fibrosis, renal tubular atrophy, increased basement membrane matrix, glomerular sclerosis and arterial intimal hyperplasia). The sections are scored according to the degree of pathological changes as follows: none, 0; mild, 1; moderate, 2; and severe, 3. The CADI is sum of the scores of all 6 sections [10]. For analysis of the immunohistochemistry-stained sections, a known positive section was used as the positive control, and α-SMA and vimentin were quantitated according to the immunohistochemistry staining range. Five fields per specimen were observed under a 100× microscope using the double-blind random method.

### Statistical methods

The SPSS 20.0 software was used for the statistical analysis and data processing. The two-sample t-test was used to compare differences in transplanted kidney function. Comparisons of multiple groups were performed using analysis of variance. The relationships between the MRI parameters and CADI scores and the expression of fibrosis markers were analyzed using Spearman’s correlation. *P* < 0.05 indicated that the difference had statistical significance.

## Results

### Conditions of the experimental animals and transplanted kidney function

**Fig. 1:**
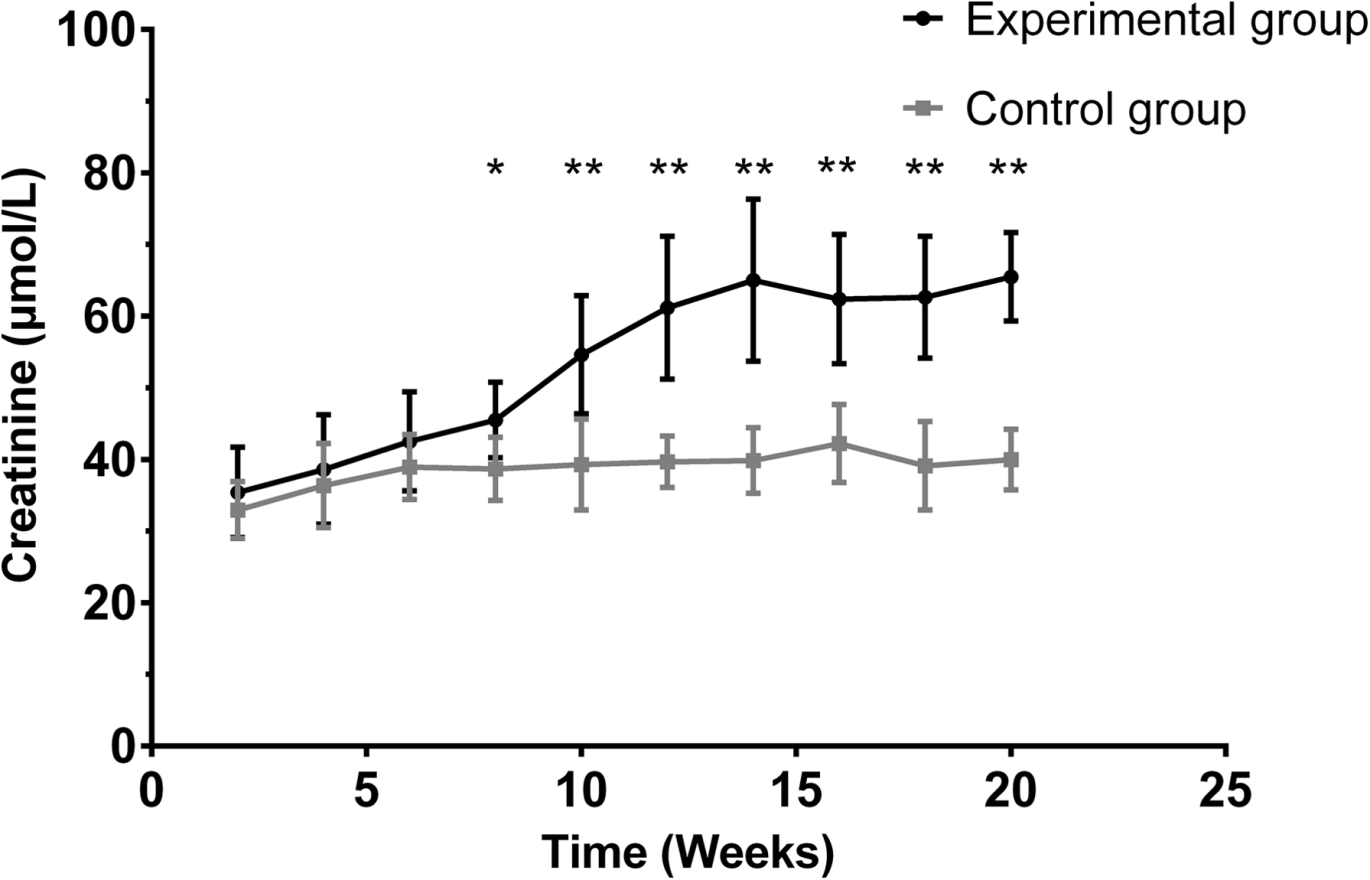
Creatinine levels at different time points in the different groups. Creatinine was measured once every 2 weeks during the experiment. The experimental group is marked in black and the control group in gray. Data are presented as the mean ± standard deviation. The creatinine level in both the experimental and control groups increased after modeling. The creatinine level in the two groups showed no significant differences until 8 weeks after modeling. (*P<0.05, **P<0.01)

No deaths occurred among the rats in the experimental and control groups. All rats completed all experiments without any adverse event. The differences in blood creatinine between the rats in the experimental and control groups were not significant until 8 weeks (P<0.05). Compared to that of the control group, the creatinine level in the experimental group gradually increased (Fig. 1).

### MRI conditions

**Fig. 2:**
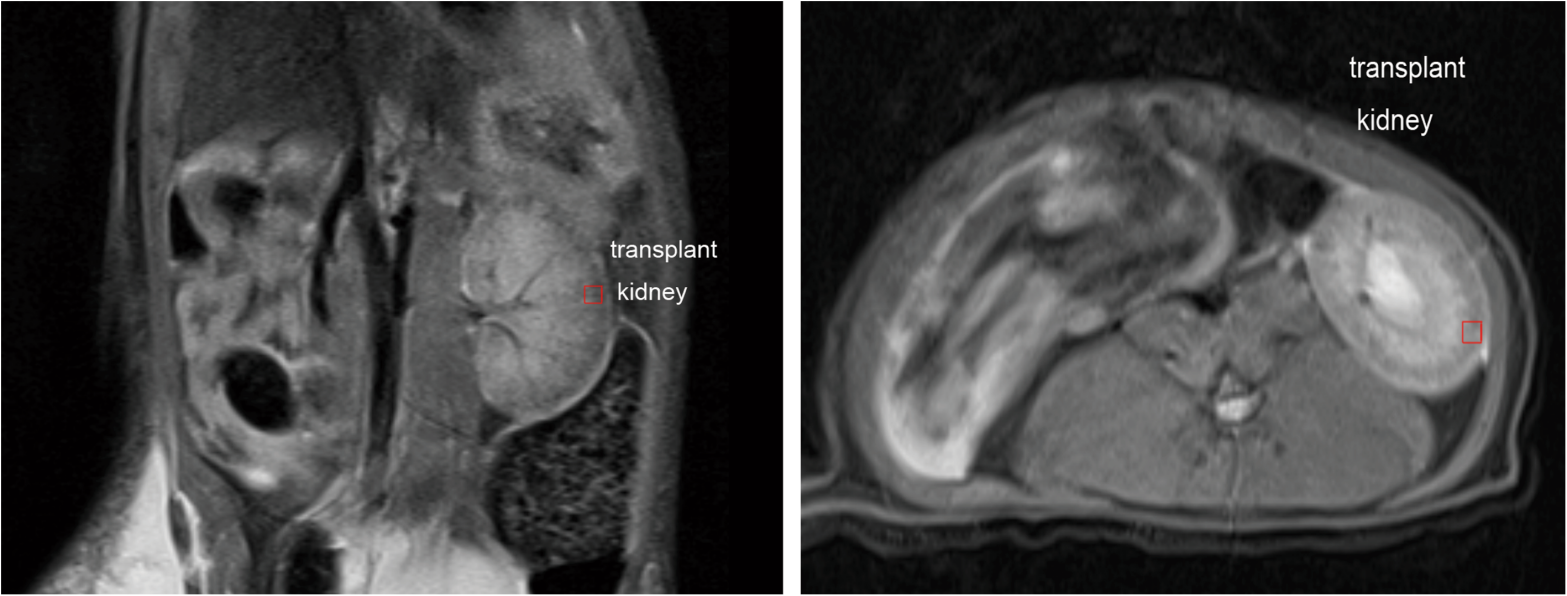
Locations of the transplanted kidneys and selection of the ROI in coronal and axial T2-weighted MRI. The transplanted kidneys were located in the original location and showed relative fixation. The regions of interest were chosen from the renal cortex randomly after avoiding blood vessels, the renal pelvis and the calyx

MRI examinations were successfully performed in all animals. The locations of the transplanted kidneys were more fixed than those of the original kidney, which might be due to the surgical procedure (Fig. 2).

#### T2-weighted images

**Fig. 3:**
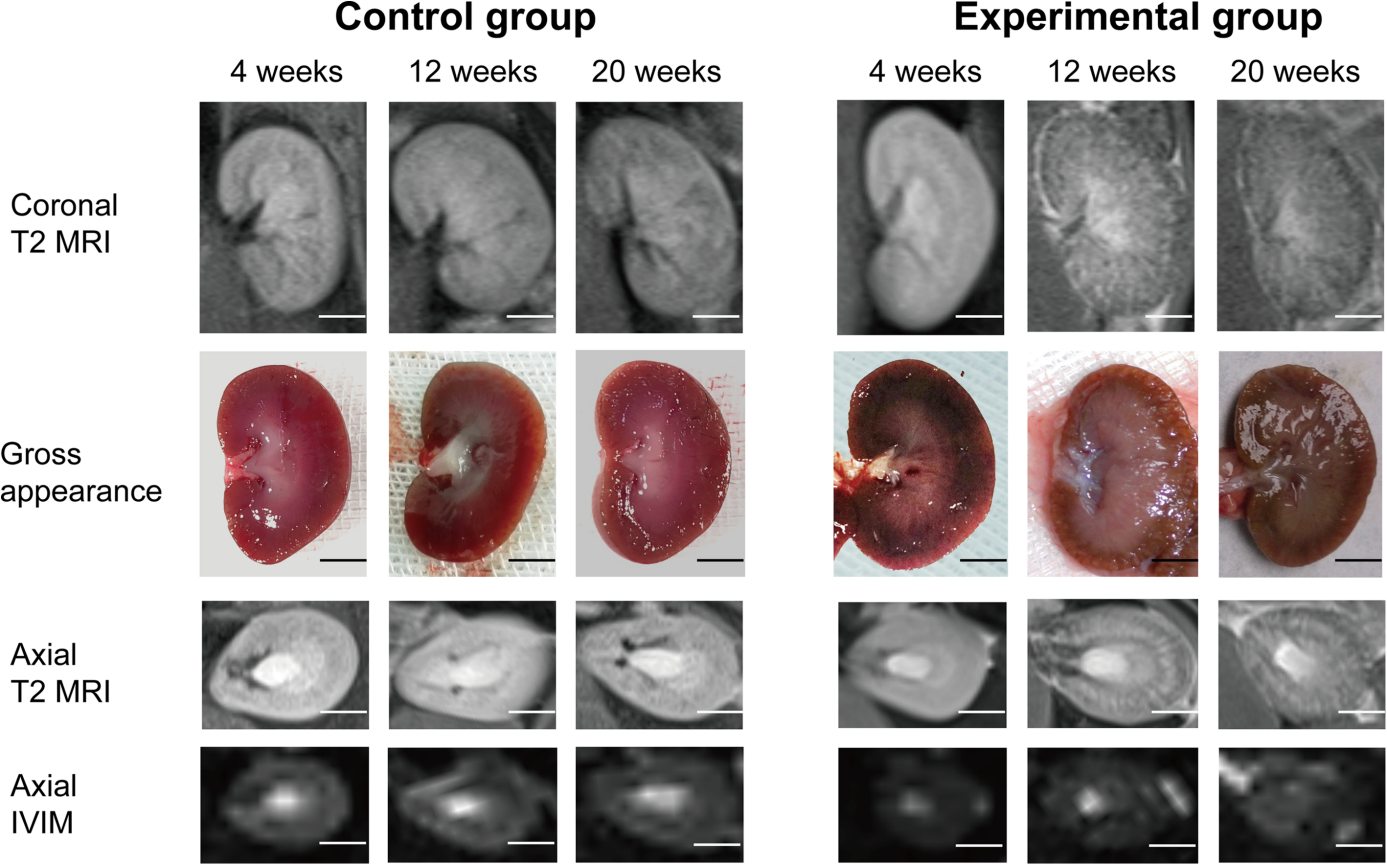
Representative T2-weighted images, IVIM and the gross appearance of the transplanted kidneys at different time points in each group. Typical coronal and axial T2-weighted images, axial IVIM (D map) and the coronal gross appearance 4, 12 and 20 weeks after modeling. In the experimental group, the signals of the cortex and medulla gradually strengthened over time, the distribution of signals in the cortex and medulla was disordered, and the boundary became vaguer. In contrast, the control group showed no significant changes over time (scale bar: 5 mm)

MRI T2 images can clearly display the size and condition of the cortex and medulla of transplanted kidneys in rats. In the transplanted kidneys of the control group rats, the renal pelvis showed an elevated T2 signal, the medulla showed a slightly higher T2 signal, the cortex showed an iso-T2 signal, and the boundary between the cortex and medulla was clear. The size of the transplanted kidneys, signal intensity and signal distribution of the control group rats at different postoperative time points did not show significant changes (*P* > 0.05). In the transplanted kidneys of the experimental group rats, morphological and signal changes occurred with the increased time after surgery, the signals of the cortex and medulla gradually strengthened, the distribution of signals in the cortex and medulla was disordered, and the boundary became vaguer. After surgery, the volume of the transplanted kidneys in the experimental group with late-stage CAD (13–20 weeks after modeling) decreased slightly, and the cortex thinned compared with that of the experimental group with early-stage CAD (0–4 weeks after modeling). The gross appearance in the control group did not show any significant changes at different time points after surgery. With the increasing time after surgery, the transplanted kidney volume in the experimental group gradually decreased, the texture gradually hardened, thinning of cortex could be observed in the longitudinal section, and small nodules were scattered in the renal parenchyma (Fig. 3).

#### DWI and IVIM

**Fig. 4:**
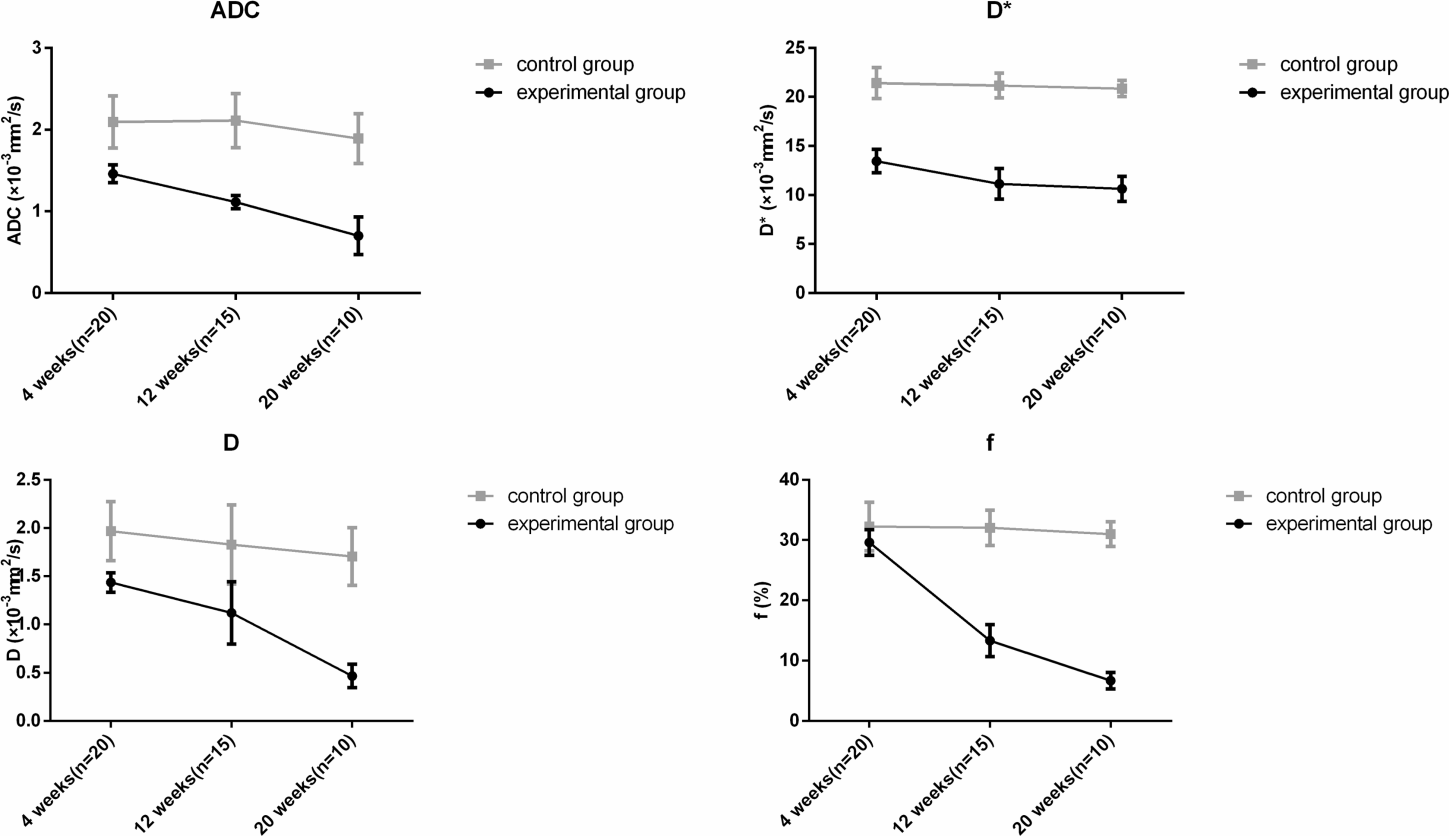
Changes in the DWI and IVIM parameters in the experimental and control groups. The ADC, D and f parameters in the experimental group decreased gradually over time (P<0.05). All parameters in the control group and D* in the experimental group showed no significant differences with the increasing postoperative time (P>0.05)

Compared with those of the control group, the postoperative ADC, D and f parameters in the experimental group with early-stage CAD decreased significantly (P<0.05). The ADC, D and f parameters in the experimental group gradually decreased with the increasing postoperative time (P<0.05). Conversely, all parameters in the control group and D* in the experimental group showed no significant differences with the increasing postoperative time (P>0.05) (Table 1 and Fig. 4).

**Table 1:** DWI and IVIM parameters of 2 groups at different stages

Time points	Parameters	Experimental group	Control group	*P* value
4 weeks (*n* = 20)	ADC (10^−3^ mm^2^/s)	1.46 ± 0.11	2.09 ± 0.32	0.000
	D* (10^−3^ mm^2^/s)	20.95 ± 2.27	21.42 ± 1.60	0.459
	D (10^−3^ mm^2^/s)	1.44 ± 0.10	1.97 ± 0.31	0.000
	f (%)	26.53 ± 2.09	32.25 ± 4.01	0.000
12 weeks (*n* = 15)	ADC (10^−3^ mm^2^/s)	1.11 ± 0.08	2.11 ± 0.33	0.000
	D* (10^−3^ mm^2^/s)	20.65 ± 0.80	21.16 ± 1.26	0.589
	D (10^−3^ mm^2^/s)	1.12 ± 0.32	1.83 ± 0.41	0.002
	f (%)	13.32 ± 2.67	32.04 ± 2.93	0.000
20 weeks (*n* = 10)	ADC (10^−3^ mm^2^/s)	0.70 ± 0.23	1.89 ± 0.31	0.000
	D* (10^−3^ mm^2^/s)	19.73 ± 0.92	20.85 ± 0.82	0.019
	D (10^−3^ mm^2^/s)	0.47 ± 0.12	1.71 ± 0.30	0.000
	f (%)	6.68 ± 1.36	31.00 ± 2.05	0.000

### Histopathological changes in the transplanted kidneys

**Fig. 5:**
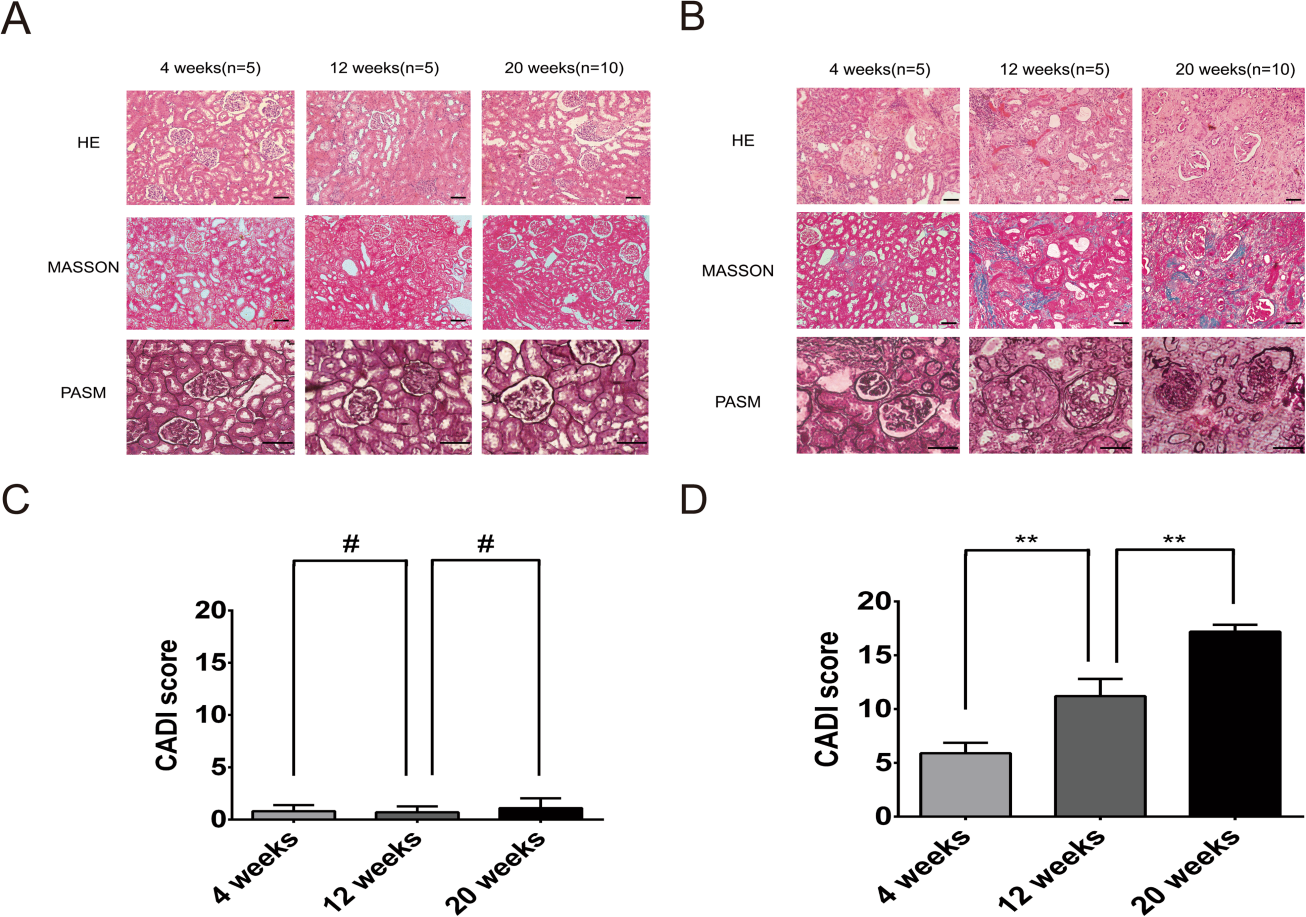
Histopathological changes in the transplanted kidneys at different postoperative time periods. **a** Pathological changes at different postoperative time periods in the control group (HE and Masson staining 200 × magnification, PASM staining 400 × magnification; scale bar: 50 μm). **b** Pathological changes at different postoperative time periods in the experimental group. The experimental group presented CAD features rather than those of the control group at 4 weeks after modeling. Additionally, the experimental group rats exhibited gradual deterioration of CAD features at different time points after surgery. **c** CADI changes in the control group. The CADI scores of the control group showed no significant differences over time (#*P* > 0.05). **d** CADI changes in the experimental group. The CADI score of the experimental group increased over time (**P<0.01)

Kidney tissue staining and immunohistochemistry sections from the different groups were observed under a light microscope. Compared with those of the control group, the experimental group already exhibited pathological changes of CAD at 4 weeks after modeling, which included mild proliferation of small arterial intima combined with a small amount of monocyte infiltration, glomerular focal sclerosis, renal tubular atrophy and interstitial fibrosis in the transplanted kidneys. The CAD pathological changes were gradually aggravated over time. Small arterial intimal hyperplasia was gradually aggravated until lumen occlusion developed, monocyte infiltration gradually increased, glomerular sclerosis, tubular atrophy, and interstitial fibrosis were gradually aggravated, and the CADI gradually increased (Fig. 5). The above findings were consistent with the pathological features of CAD and indicated that the CAD rat model was successfully established.

**Fig. 6:**
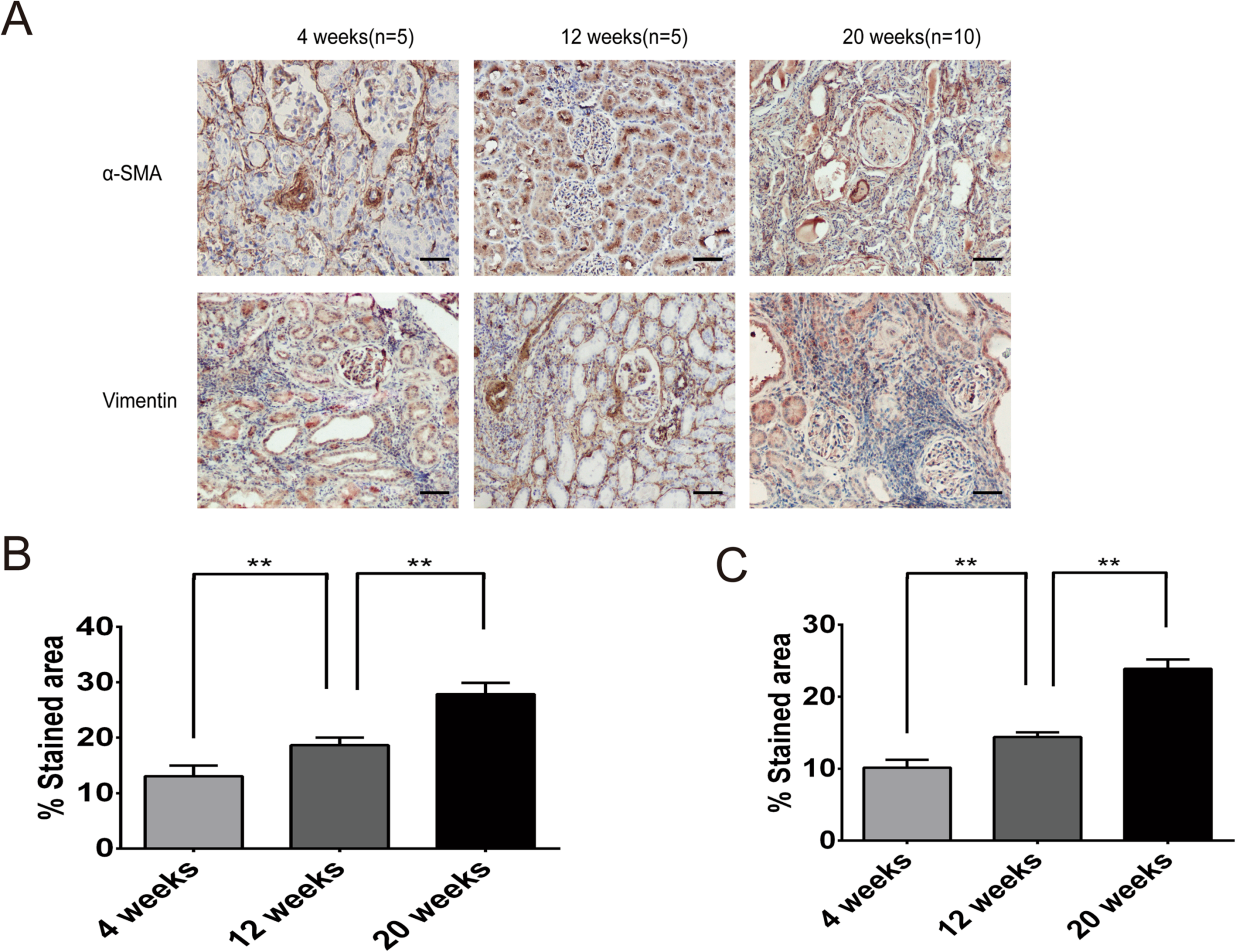
Postoperative α-SMA and vimentin expression in the control group. **a** α-SMA and vimentin expression in the transplanted kidneys at different postoperative time points in the experimental group (400× magnification, scale bar: 50 μm). **b** α-SMA expression levels at different postoperative time points in the experimental group. **c** Vimentin expression levels at different postoperative time points in the experimental group. (**P<0.01)

The graft interstitial α-SMA and vimentin expression levels in the experimental group gradually increased over time. The quantitative analysis showed that the differences were statistically significant (P<0.05) (Fig. 6).

### Relationships between MRI-related indicators and the CADI and fibrosis markers for the transplanted kidneys in the experimental group

The linear correlation analysis showed that the DWI parameter ADC (*r* = − 0.849, *P* < 0.01; *r* = − 0.874, *P* < 0.01; and *r* = − 0.875, *P* < 0.01) and IVIM parameters D (*r* = − 0.665, *P* < 0.01; *r* = − 0.702, *P* < 0.01; and *r* = − 0.764, *P* < 0.01) and f (*r* = − 0.941, *P* < 0.01; *r* = − 0.905, *P* < 0.01; *r* = − 0.853, *P* < 0.01) were negatively correlated with the CADI and the α-SMA and vimentin expression levels, respectively (Table 2).

**Table 2:** Association of renal cortical MRI parameters with CADI and fibrosis markers

	ADC (10^−3^ mm^2^/s)	D* (10^−3^ mm^2^/s)	D (10^− 3^ mm^2^/s)	f (%)
correlation coefficient with CADI	−0.849**	−0.339	−0.665**	−0.941**
correlation coefficient with α-SMA	−0.874**	− 0.335	− 0.702**	−0.905**
correlation coefficient with vimentin	−0.875**	−0.033	− 0.764**	−0.853**

**P < 0.01

## Discussion

The clinical manifestation of CAD is progressive reduction of transplanted kidney function combined with hypertension and proteinuria. The pathological features are renal interstitial fibrosis, renal tubular atrophy and vascular changes. The gradual progression of CAD ultimately results in graft fibrosis and causes graft failure. Graft fibrosis is the most important reason for graft failure at the late stage. This occult pathological progression usually proceeds in a slow and dynamic manner that eventually results in increased blood creatinine in kidney transplant recipients. When elevation of blood creatinine is discovered, injury of the transplanted kidney has already reached the irreversible stage [11]. CAD is an important reason for graft failure. Currently, specific treatment methods for CAD are lacking. Animal studies have suggested that early intervention can prevent CAD progression and maintain graft function [12]. Effective monitoring and adoption of corresponding treatment will prolong the time before recipients develop graft failure, which will greatly help promote and improve the long-term survival of transplanted organs [13].

The current gold standard for a CAD diagnosis is biopsy of the transplanted kidneys. Currently, a protocol biopsy is performed for early discovery and diagnosis of CAD in clinical practice. However, usually patients are reluctant to accept this invasive examination. The pathological results of the transplanted kidney biopsy are influenced by the locations and numbers of biopsy samples. In addition, pathological results have greater subjectivity and are difficult to quantitate [14]. Therefore, ultrasonography has become a routine monitoring method for transplanted kidneys after surgery [15]. We previously confirmed the application value of contrast-enhanced ultrasound (CEUS) for the early diagnosis of CAD in rats [16]. However, the technical requirements of CEUS for operators are high, and its specificity is relatively poor [17, 18]. As an imaging examination that is easy to operate, has high resolution, and does not involve radiation, MRI has been widely applied in clinical practice. MRI not only can provide high-resolution morphological and unilateral kidney information but also can reflect changes in the living condition at the molecular level [19].

DWI can detect the motion of water molecules in tissues to reflect the pathophysiological features of tissue structures. In addition to providing diffusion motion information for water molecules, the IVIM model can also provide microcirculation perfusion effect information [19, 20]. To evaluate the influence of the cold ischemia time on transplanted kidneys, Rheinheimer et al. [21] used IVIM to quantitatively evaluate diffusion and perfusion information from transplanted kidneys. They found that the ADC, D and f values of transplanted kidneys were significantly lower than those of healthy controls. In addition, the reduction of these parameters was even more obvious in transplanted kidneys with a prolonged cold ischemia time. They concluded that the changes in IVIM parameters were associated with the cold ischemia time. Liang et al. [19] applied IVIM to evaluate contrast-induced acute kidney injury in a rat model. The results showed that the ADC and D values decreased progressively with the progression of renal injury. Therefore, they concluded that IVIM could effectively monitor the progression of contrast-induced acute kidney injury in rats. The above studies provided inspiration to further discuss the application of IVIM for the early diagnosis of CAD and monitoring of the graft fibrosis stage.

We found that the ADC, D and f values were significantly decreased before any significant difference in creatinine was detected between the rats in the early-stage CAD and control groups. The pathological examination of the transplanted kidneys at the same period confirmed that the experimental group already exhibited corresponding CAD pathological changes. The ADC parameter value reflects the speed of the diffusion motion of water molecules in tissues. Traditional ADC values contain the double influences of the diffusion and perfusion effects. The D value reflects the actual diffusion of water molecules. D* reflects perfusion-related diffusion and may be associated with the average lengths of the renal tubules and the liquid flow rate. The f value reflects the degree of abundance of capillaries in tissues [22].

The ADC value is closely associated with renal function and the degree of fibrosis. The ADC value decreases with decreasing renal function and aggravation of fibrosis [23], which may be associated with the accumulation of myofibroblast (MFB) products [24]. MFB is the most important effector cell causing graft fibrosis via the extracellular matrix (ECM) [25]. The accumulated ECM can block diffusion of water molecules, causing reduction of the ADC and IVIM indicators [26, 27]. Immunohistochemistry staining showed that compared with those of the control group, the experimental group exhibited accumulation of MFB markers (α–SMA and vimentin) at the early stage of CAD. This result also confirmed the association between the reduction of ADC and IVIM indicators and the accumulation of MFB products.

The experiments showed that the ADC, D and f values in IVIM were decreased at the early stage in the experimental group, whereas D* did not significantly differ between the experimental group with early-stage CAD and the control group. This result was similar to the findings of Hennedige et al. in an obstructive renal fibrosis model and might be associated with the uneven distribution between the renal blood flow and renal tubular flow caused by fibrosis [22]. The results of this study showed that the reduction of IVIM indicators occurred earlier than the elevation of creatinine, which might be associated with renal function compensation in the early stage of CAD. Thus, the application value of IVIM for the early discovery of CAD was confirmed.

Analysis of the pathology of the rat CAD model in the experimental group at different postoperative time periods showed that the pathological progression of CAD developed gradually over time, the CADI score gradually increased, and the corresponding IVIM indicators also gradually changed. The linear correlation analysis showed that with the increasing CADI score, the ADC, D and f values of the transplanted kidneys gradually decreased, whereas D* showed no significant correlation. These results indicated that IVIM imaging could indirectly reflect CAD progression in the transplanted kidneys to some extent. The significant correlation between IVIM indicators and α-SMA/vimentin also provided a possible reason for its reflection of CAD progression; the CAD progression condition is indirectly reflected through the level of graft fibrosis, which is an important CAD presentation. These results provide a theoretical basis for application of IVIM for evaluation of the graft fibrosis stage.

The subject of this study was the rat CAD model, which ensured homogeneity of the research subjects and comprehensive evaluation of the transplanted kidney specimens. The experimental design also minimizes the use of experimental animals under the premise of ensuring the quality of the experiment. The further human assessment is needed before clinical use. Due to limitation of the condition for the experiment, we used a clinical head coil rather than a dedicated animal coil. Additionally, the correlation between the IVIM parameters and pathological changes might be a coincidence due to the limited sample size. The other limitation is that the sensitivity and specificity of these markers are not tested in this study thus a biopsy is still necessary for the diagnosis. However, this technology can increase the diagnostic level of subclinical injury of transplanted kidneys and the level of disease progression of the graft, thereby providing an alternative diagnostic method for patients for whom a biopsy is contraindicated.

In summary, this study confirmed the feasibility of IVIM for the early diagnosis of CAD in rats and showed a correlation between IVIM indicators and the degree of chronic pathological injury in transplanted kidneys and the fibrosis markers α-SMA and vimentin. These results provided a basis for application of IVIM for evaluation of the degree of chronic pathological injury and graft fibrosis in CAD.

## Conclusion

IVIM imaging can detect CAD earlier than changes in creatinine and correlates with the degree of fibrosis in grafts.

## Data Availability

The datasets used and/or analyzed during the current study are available from the corresponding author on reasonable request.

## Abbreviations

ADC: Apparent diffusion coefficient
CAD: Chronic allograft damage
CADI: Chronic allograft damage index
CEUS: Contrast-enhanced ultrasound
DWI: Diffusion weighted imaging
ECM: Extracellular matrix
ESRD: End-stage renal disease
FOV: Field of view
ICC: Intraclass correlative coefficient
IVIM: Intravoxel incoherent motion
MFB: Myofibroblast
PASM: Periodic acid-silver methenamine
ROI: Region of interest
TE: Echo time
TR: Repetition time
TSE: Turbo spin-echo
α-SMA: α-smooth muscle actin
